# Moderate hypothermia circulatory arrest as a brain-protective strategy for type A aortic dissection

**DOI:** 10.1093/icvts/ivae166

**Published:** 2024-10-03

**Authors:** Hodaka Wakisaka, Shunta Miwa, Yuji Matsubayashi, Yotaro Mori, Junghun Lee, Kenichi Kamiya, Noriyuki Takashima, Tomoaki Suzuki

**Affiliations:** Department of Cardiovascular Surgery, Shiga University of Medical Science, Shiga, Japan; Department of Cardiovascular Surgery, Shiga University of Medical Science, Shiga, Japan; Department of Cardiovascular Surgery, Shiga University of Medical Science, Shiga, Japan; Department of Cardiovascular Surgery, Shiga University of Medical Science, Shiga, Japan; Department of Cardiovascular Surgery, Shiga University of Medical Science, Shiga, Japan; Department of Cardiovascular Surgery, Shiga University of Medical Science, Shiga, Japan; Department of Cardiovascular Surgery, Shiga University of Medical Science, Shiga, Japan; Department of Cardiovascular Surgery, Shiga University of Medical Science, Shiga, Japan

**Keywords:** Acute type A aortic dissection, Brain protection, Stroke, Moderate hypothermia circulatory arrest, No cerebral perfusion

## Abstract

**OBJECTIVES:**

Brain-protective strategies for acute type A aortic dissection (TAAD) remain controversial. Moderate hypothermia circulatory arrest (MHCA) without cerebral perfusion is not commonly used. However, we aimed to assess its safety and efficacy in 358 patients who underwent hemiarch replacement with MHCA for acute type A aortic dissection at our institution from August 2012 to August 2022.

**METHODS:**

Clinical outcomes were compared according to circulatory arrest time [≤15 min (S group, *n* = 52) vs ≥16 min (L group, *n* = 306)]. The primary outcome was postoperative stroke.

**RESULTS:**

The S group had more older patients (72.5 vs 68.8 years; *P* = 0.04), a greater incidence of carotid artery malperfusion (21% vs 11%; *P* = 0.043) and a lower body mass index (21.7 vs 23.6 kg/m^2^; *P* < 0.01) and hemodynamic instability (3.8% vs 16%; *P* = 0.02) than the L group. The incidence of postoperative stroke (7.7% vs 12%; *P* = 0.33) and the rate of 30-day mortality (5.8% vs 6.5%; *P* = 0.83) did not significantly differ between groups. After adjusting for all potential confounding factors pre- and intraoperatively, there was no significant difference in postoperative outcomes between groups.

**CONCLUSIONS:**

MHCA alone for TAAD had comparable postoperative outcomes with circulatory arrest times under and over 15 min. However, longer arrest times were associated with a higher risk of stroke.

## Graphical Abstract

**Figure:**
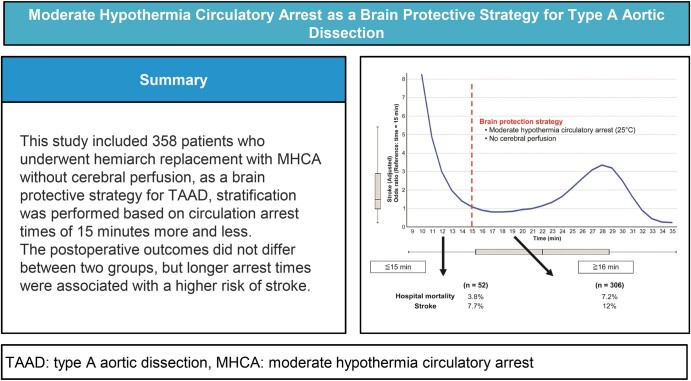

## INTRODUCTION

Acute type A aortic dissection (TAAD) is a catastrophic and life-threatening disease. According to previous studies, the rate of postoperative mortality and stroke in TAAD is 11–16.9% and 9.5–14.4%, respectively [1–4]. The clinical outcomes of surgery have improved in recent decades. Nevertheless, neurological complications are inevitable in this disease [5]. Brain protection during intraoperative circulatory arrest is essential, and it affects postoperative outcomes such as neurological complications and survival.

In the 1970s, Griepp *et al.* introduced the use of profound hypothermia circulatory arrest (HCA) to improve brain ischaemic outcomes by suppressing its metabolic demands. Moreover, this strategy has been used in surgery for TAAD to prevent organ ischaemia [6]. Patients with HCA without cerebral perfusion commonly present with profound hypothermia (<14°C) or deep hypothermia (14.1–20°C). Previous reports have revealed that the safe upper limit for deep hypothermia circulatory arrest (DHCA) is about 30 min [7, 8]. In HCA, a lower temperature can have protective effects on organs, particularly the brain, and can increase the permissible period of circulatory arrest. However, it increases the risk of various conditions, such as a longer cardiopulmonary bypass (CPB) time, coagulation disorders and systemic inflammatory response syndrome (SIRS). Hence, the advantages and disadvantages of this strategy must be balanced considering the temperature, circulatory arrest time and organ protection. A recent consensus considers using cerebral perfusion, antegrade cerebral perfusion (ACP), or retrograde cerebral perfusion (RCP) to increase the temperature at circulatory arrest, but this remains controversial [9]. There are only a few reports about the use of moderate hypothermia (20.1–28°C) circulatory arrest [moderate hypothermia circulatory arrest (MHCA)] without cerebral perfusion because the time limit for circulatory arrest is shorter and the risk of cerebral complications is higher [10]. The duration of the protective effect of MHCA on the brains of humans in real-world settings is unknown. However, it has been reported in calculation and biological experiments using a porcine model [11, 12].

The current study aimed to identify the clinical performance of MHCA alone as a brain-protective strategy for TAAD in hemiarch replacement and whether circulatory arrest time with such a strategy in real practice is longer than previously reported.

## PATIENTS AND METHODS

### Ethical statement

The Institutional Review Board of Shiga University of Medical Science (R2021-167) approved this study on 15 February 2022. The need for a written informed consent was waived because due to the retrospective nature of this study. Relevant identifiers were stripped from the data.

### Patients and study design

**Figure 1:**
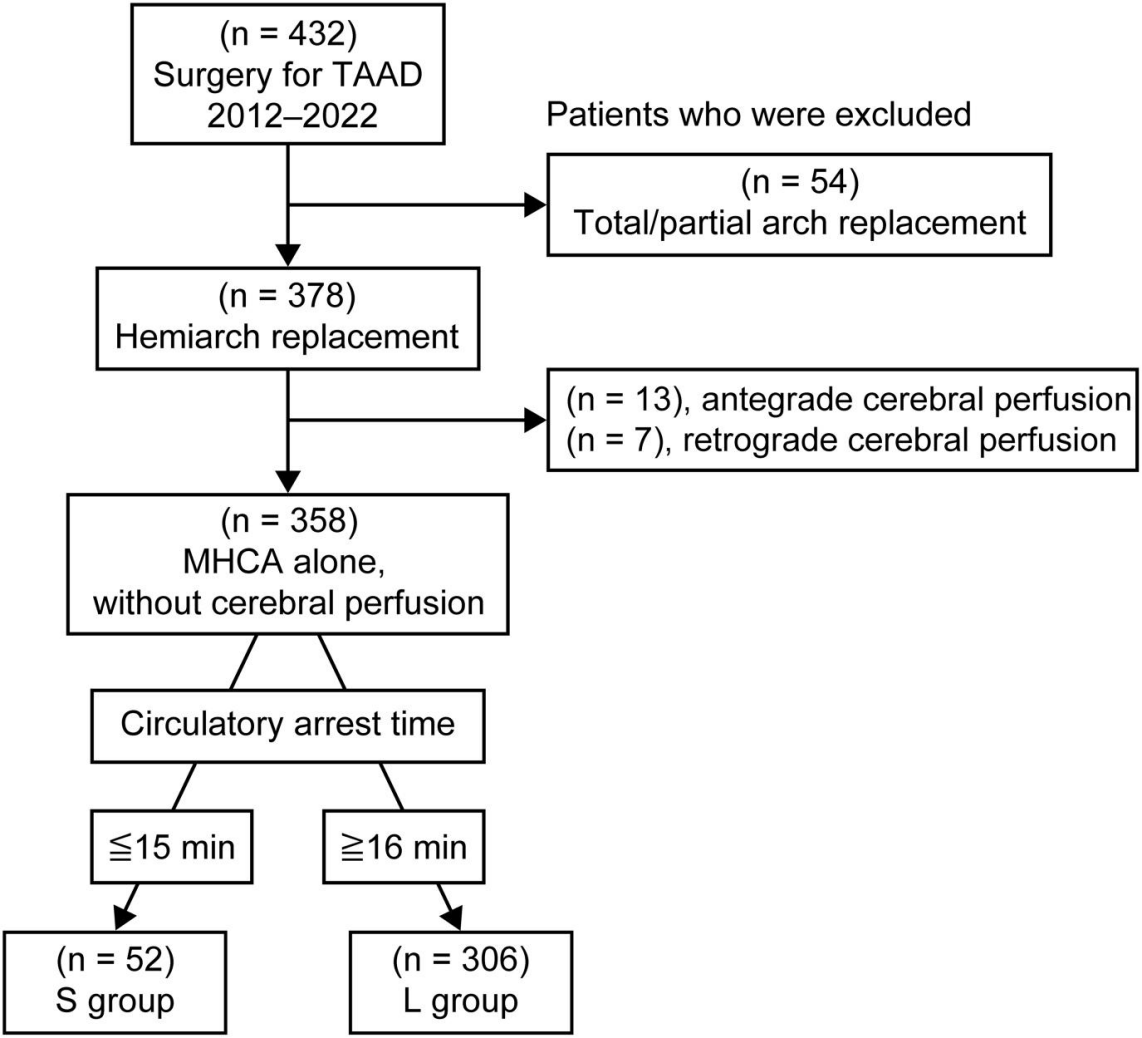
Patient selection. MHCA: moderate hypothermic circulatory arrest; TAAD: type A aortic dissection.

We retrospectively reviewed a database from August 2012 to August 2022 to identify patients who underwent hemiarch replacement with MHCA without cerebral perfusion for TAAD at Shiga University of Medical Science. In total, 432 patients underwent emergency surgical repair for TAAD. Among them, 378 underwent hemiarch replacements, and 54 had total arch or partial arch replacements. After excluding 13 patients with ACP and 7 with RCP, 358 patients were found to be eligible in this study. We compared the clinical outcomes of patients with a short circulation arrest time (≤15 min, S group, *n* = 52) and those with a long circulation arrest time (≥16 min, L group, *n* = 306). The reason for this borderline was the calculated safe duration of HCA at 25°C was taken about 15 min as a reference [11] (Fig. 1).

The primary outcome was postoperative stroke. The secondary outcomes included early mortality and other postoperative complications.

Moderate hypothermia was defined as a tympanum temperature of 20.1–28°C during circulatory arrest based on a previous report [13]. Postoperative stroke was defined as a permanent neurological deficit with evidence of cerebral infarction on computed tomography scan or magnetic resonance imaging that was confirmed by a neurologist. Preoperative conditions were not considered, and the onset time was any time during the perioperative period. Postoperative stroke also included patients with evident signs of severe cerebral damage or oedema who died but did not undergo clinical neurological assessment postoperatively. Haemodynamic instability was defined as preoperative hypotension (systolic blood pressure <60 mmHg, which is the borderline blood pressure that can cause brain damage) attributed to bleeding, cardiac tamponade, malperfusion, myocardial ischaemia or infarction, or acute congestive heart failure. The definitions for the other components were based on the STS-ACSD criteria [14].

### Surgical technique

Previous studies have shown the details of the surgical technique for TAAD at our facility [15, 16]. All approaches were used in median sternotomy. The arterial cannulation site (femoral or axillary artery vs ascending aorta) was selected based on the patient’s condition. The common site was the femoral artery. In all cases, whole-body cooling and a tympanum temperature of 25°C are a point for circulation arrest. If the core temperature (bladder or rectal) did not decrease to 30°C, one should wait until it drops to <30°C. The ascending aorta was opened with circulatory arrest. Retrograde cold blood cardioplegia was inserted directly into the coronary sinus via right atriotomy intermittently for myocardial protection. If there was an intimal tear in the ascending aorta or proximal transverse aorta or no tear in the transverse aorta on direct visualization, only hemiarch replacement was performed using an open distal anastomosis with MHCA without cerebral perfusion. ACP or RCP was additionally performed if cerebral oxygenation based on oximetry monitoring with INVOS 5100C (Somanetics, Troy, MI, USA) significantly decreased or if the arrest time was likely to exceed 30 min because of the complexity of the distal anastomosis. After the distal repair with 3-0 monofilament continuous suturing with Teflon felt strips (DuPont, Wilmington, DE, USA) reinforced around the perimeter, RCP was performed for about 30 seconds to prevent embolization and facilitate deairing, not for cerebral protection. Then, CPB was restarted via the prosthesis’s side branch, and rewarming was started. We resumed coronary perfusion after the proximal anastomosis. Performing hemiarch or total/partial arch replacement and whether to use with cerebral perfusion was based on the surgeon’s discretion.

### Statistical analysis

Stratification was performed based on circulation arrest times. Continuous variables were presented as mean with standard deviation or medians with interquartile ranges. Categorical data were expressed using frequency and percentage. As all continuous variables had skewed distributions, Mann–Whitney *U* tests were employed. Categorical variables were analysed using χ^2^, and Fisher’s exact tests for frequencies of <5. The logistic regression model was utilized to estimate the adjusted odds ratio with 95% confidence interval for the association between circulation arrest time and postoperative complications. The multivariable logistic regression model was performed with adjustments for all potential confounding factors (Table 1), the cannulation site and concomitant procedures. To assess the nonlinear association between the duration of MHCA and postoperative stroke, a multivariable logistic regression analysis was performed using the cubic spline. The nonlinear association between predictor and outcome was expressed as a spline curve combining cubic polynomials and linear terms in the cubic spline. Outliers were added using a box plot.

**Table 1:** Preoperative characteristics of the patients

Characteristics	All patients	S group (≤15 min)	L group (≥16 min)	*P* value
	(*n* = 358)	(*n* = 52)	(*n* = 306)	
Age (years), mean ± SD	69.3 ± 12.9	72.5 ± 13.0	68.8 ± 12.8	0.04
>80 years	90 (25)	20 (38)	70 (23)	0.02^a^
Body mass index (kg/m²), mean ± SD	23.3 ± 4.0	21.7 ± 3.7	23.6 ± 4.0	<0.01
Male sex, *n* (%)	195 (54.5)	26 (50)	169 (55)	0.48
Hypertension, *n* (%)	243 (67.9)	34 (65)	209 (68)	0.68
Diabetes mellitus, *n* (%)	34 (9.5)	3 (5.8)	31 (10)	0.32
Dyslipidaemia, *n* (%)	101 (28.2)	16 (31)	85 (28)	0.66
Smoking history, *n* (%)	143 (39.9)	19 (37)	124 (41)	0.59
Old cerebrovascular accident, *n* (%)	37 (10.3)	6 (12)	31 (10)	0.76
Malfan syndrome, *n* (%)	17 (4.7)	1 (1.9)	16 (5.2)	0.3
Renal insufficiency, *n* (%)	38 (10.6)	3 (5.8)	35 (11)	0.22
Lung disease, *n* (%)	49 (13.7)	11 (21)	38 (12)	0.09
Previous history of cardiac surgery, *n* (%)	11 (3.1)	1 (1.9)	10 (3.3)	0.6
DeBakey type II, *n* (%)	72 (20.1)	11 (15)	61 (20)	0.84
Malperfusion, *n* (%)	74 (20.1)	14 (27)	60 (20)	0.23
Coronary, *n* (%)	11 (3.1)	2 (3.8)	9 (2.9)	0.73
Carotid, *n* (%)	45 (12.6)	11 (21)	34 (11)	0.043^a^
Visceral, renal, *n* (%)	38 (10.6)	9 (17)	29 (9.4)	0.09
Extremities, *n* (%)	30 (8.4)	5 (9.6)	25 (8.2)	0.73
Aortic valve insufficiency, *n* (%)	44 (12.3)	7 (13)	37 (12)	0.78
Neurological deficient, *n* (%)	67 (18.7)	8 (15)	59 (19)	0.51
Haemodynamic instability, *n* (%)	51 (14.2)	2 (3.8)	49 (16)	0.02^a^
Cardiopulmonary resuscitation, *n* (%)	22 (6.1)	1 (1.9)	21 (6.9)	0.17

SD: standard deviation.

a *P*-value based on the Fisher’s exact test.

Data were analysed using the Statistical Package for the Social Sciences version 25.0 and R 4.3.1. A *P* value of <0.05 was considered statistically significant.

## RESULTS

### Characteristics of the patients

In total, 358 patients underwent hemiarch repair with MHCA without cerebral perfusion as a brain-protective strategy for TAAD from 2012 to 2022. Table 1 shows the baseline characteristics of the two groups. The S group had a higher proportion of older patients (72.5 vs 68.8 years; *P* = 0.04) and a lower body mass index (21.7 vs 23.6 kg/m^2^; *P* < 0.01) and haemodynamic instability (3.8% vs 16%, *P* = 0.02) than the L group. The other preoperative characteristics or dissection-related status did not differ between the two groups.

### Operative and postoperative data

Table 2 shows the operative data. The cannulation site and concomitant procedures did not significantly between the S and L groups. The L group had a longer circulatory arrest time than the S group. Thus, it also had a longer CPB and cross-clamp times. Table 3 shows the postoperative data. The 30-day mortality and stroke rates were 5.8% and 7.7% in the S group and 6.5% and 12% in the L group, respectively. The L group had higher 30-day mortality and stroke rates than the S group but were not significantly different (*P* = 0.83 and 0.33, respectively). The other outcomes, such as spinal ischaemia, new haemodialysis, reoperation for bleeding, tracheostomy and gastrointestinal complications, were not different between the two groups. Table 4 shows the multivariable logistic regression model with adjustments for all potential confounding factors (Table 1), the cannulation site, and concomitant procedures. There were no differences in all outcomes after adjustment.

**Table 2:** Operative data

	All patients	S group (≤15 min)	L group (≥16 min)	*P* value
	(*n* = 358)	(*n* = 52)	(*n* = 306)	
Cannulation site				
Ascending aorta	22 (6.1)	2 (3.8)	20 (6.5)	0.46
Axillary artery	56 (15.6)	8 (15)	48 (16)	0.96
Femoral artery	272 (76.0)	40 (77)	232 (76)	0.86
Axillary + femoral artery	8 (2.2)	2 (3.8)	6 (2.0)	0.4
Duration of procedure (min), median (IQR)				
Cardiopulmonary bypass time	97 (88–112)	88 (79–98)	100 (90–113)	<0.01
Cross-clamp time	46 (39–55)	36 (32–42)	48 (42–57)	<0.01
Circulatory arrest time	20 (17–23)	14 (13–14)	21 (18–23.5)	<0.01
Nadir temperature (Celsius), median (IQR)				
Tympanic	24.5 (23.4–25.0)	24.5 (23.0–25.0)	24.5 (23.4–25.0)	0.61
Bladder or rectal	27.2 (25.7–28.5)	27.2 (25.8–28.4)	27.2 (25.6–28.5)	0.68
Concomitant surgery				
Aortic root replacement	5 (1.4)	1 (1.9)	4 (1.3)	0.73
Aortic valve repair or replacement	23 (6.4)	4 (7.7)	19 (6.2)	0.69
Coronary bypass	26 (7.3)	3 (5.8)	23 (7.5)	0.65
Lower limb bypass	16 (4.5)	1 (1.9)	15 (4.9)	0.34

IQR, interquartile range.

**Table 3:** Postoperative data

	All patients	S group (≤15 min)	L group (≥16 min)	*P* value
	(*n* = 358)	(*n* = 52)	(*n* = 306)	
30-day mortality	23 (6.4)	3 (5.8)	20 (6.5)	0.83
Hospital mortality	24 (6.7)	2 (3.8)	22 (7.2)	0.37
Stroke	42 (11.7)	4 (7.7)	38 (12)	0.33
Spinal ischaemia	8 (2.2)	1 (1.9)	7 (2.3)	0.87
New haemodialysis	24 (6.7)	2 (3.8)	22 (7.2)	0.37
Reoperation for bleeding	52 (14.5)	5 (9.6)	47 (15)	0.28
Tracheostomy	28 (7.8)	2 (3.8)	26 (8.5)	0.25
GI complication	12 (3.4)	0	12 (3.9)	0.15
Postoperative IABP and PCPS	9 (2.5)	2 (3.8)	7(2.3)	0.51

GI: gastrointestinal; IABP: intra-aortic balloon pumping; PCPS: percutaneous cardiopulmonary support.

**Table 4:** Multivariable logistic regression analysis of outcomes according to circulatory arrest time ≥16 vs ≤15 min

Variables	Hazard ratio	95% Confidence interval	*P* value
30-day mortality	0.7	0.12–5.30	0.7
Hospital mortality	1.54	0.20–21.44	0.71
Stroke	0.65	0.13–4.12	0.62
Spinal ischaemia	1.03	0.01–165.17	0.99
New haemodialysis	0.67	0.14–5.17	0.65
Reoperation for bleeding	0.68	0.20–2.73	0.56
Tracheostomy	0.93	0.19–7.17	0.94

## DISCUSSION

Some studies have shown that brain-protective strategies with cerebral perfusion can reduce the risk of mortality and stroke compared with HCA alone [6, 7]. Several studies have compared cerebral protection performance based on information from different databases. According to the STS database, O'Hara *et al.* reported that the mortality rates of HCA, ACP and RCP were 17.5%, 15.8% and 16.5% in TAAD and 13.0%, 12.5% and 11.2% in stroke, respectively [4]. The European Society of Cardiology guidelines on aortic disease recommend adopting the selective ACP technique, which reduces the risk of stroke (class IIa, level B) [17]. Cerebral perfusion may prevent cerebral ischaemia and extend the time limit for circulatory arrest. However, ACP is associated with a risk of embolism and cerebral vessel injury when manipulating the cerebral vessels because of the fragility of the dissected vessels, particularly in TAAD. In RCP, cerebral oxygenation is suboptimal [18]. By contrast, HCA alone has advantages. For example, it is simple to operate and can provide a bloodless field. Therefore, the optimal strategy for brain protection against TAAD remains controversial.

Some studies have shown that MHCA is safe and effective compared with DHCA, with ACP or RCP [19–21]. Recently, to reduce the weakness of hypothermia, such as a longer CPB time, coagulation disorders and SIRS, there has been a shift towards using MHCA in conjunction with ACP or RCP, thereby allowing surgeons to extend the safe duration of circulatory arrest at warmer temperatures. Similarly, if HCA strategies alone are safe when increasing temperature during circulatory arrest, it could overcome the weakness of hypothermia and prevent the risk and hassle of cerebral perfusion, which can improve postoperative outcomes. Svensson *et al.* reported an increased incidence of stroke after 40 min of DHCA alone and a significant increase in the mortality rate at 65 min after the procedure [22]. Yan *et al.* showed that DHCA alone affords approximately 20–30 min of safe HCA time and MHCA for 10–20 min [13]. There are only a few reports about the use of MHCA alone, without cerebral perfusion [10], and its safety has not been established. McCullough *et al.* showed that the calculated safe duration of HCA at 18°C was <26 min. However, it is only 14 min at 25°C [11]. In biological experiments using the porcine model, the estimated permissible period of HCA was only 11 min at 24°C [12]. However, it is unknown how long it can protect the brain at MHCA alone in real-world settings for humans.

**Figure 2:**
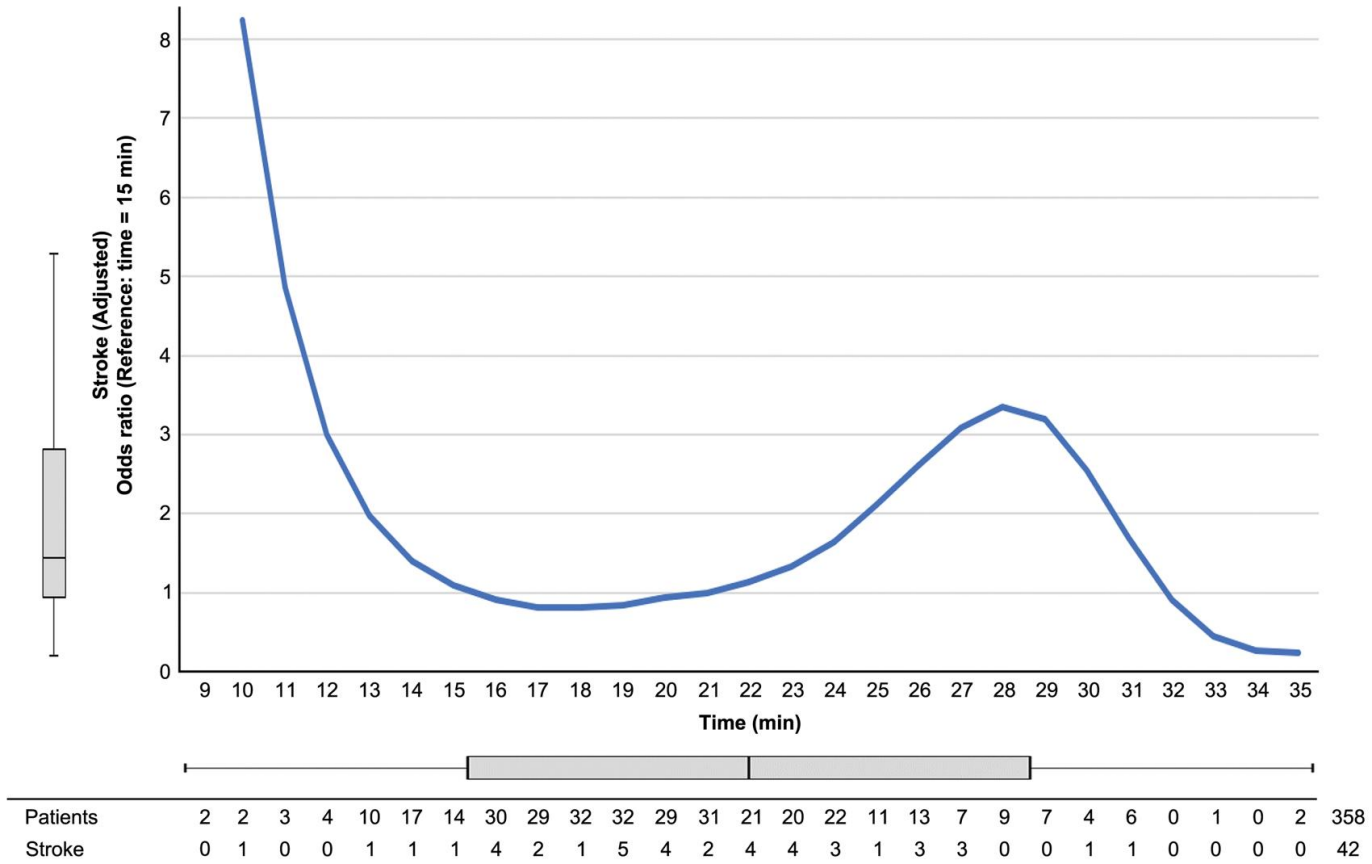
Nonlinear association between MHCA duration and the multivariable adjusted postoperative stroke odds ratio. MHCA: moderate hypothermic circulatory arrest.

This study showed no difference in terms of postoperative outcomes if the circulatory arrest time at 25°C exceeds 15 min. Hence, a longer circulatory arrest time at this temperature may be more acceptable than those in the past. By contrast, nonlinear associations (Fig. 2) showed a trend towards an increased risk of stroke after approximately 23 min. Patients with longer circulatory arrest times and a higher stroke risk were excluded from this study because of the use of cerebral perfusion. Hence, this could be the reason why there was a lower stroke risk after 30 min. Regardless of whether this method is recommended, it is useful to know that brain damage is reduced up to this time even if establishing brain perfusion requires time using ACP in moderate hypothermia.

When reporting the temperature of cerebral protection, it is essential to indicate the specific origin of the temperature. We used tympanic temperature as the monitor of brain temperature. Previous studies have reported that oesophageal temperature best reflects brain temperature, and a recent consensus recommends using pharyngeal temperature [13]. Stone *et al.* reported the difference between brain and tympanic temperatures during circulatory arrest had an error of 2.6°C. However, the errors on oesophageal and pharyngeal temperature were 1.9°C and 2.1°C respectively, indicating that the errors were not large on tympanic temperature [23]. The tympanic temperature is easy to insert even in emergencies. We not only used tympanic temperature but also core temperature (bladder or rectal) as the criteria for circulation arrest as we believe that a high core temperature provides inadequate protection of the spinal cord and visceral organs.

Stroke in TAAD is challenging to evaluate and compare because it is caused by not only brain protection but also a combination of various factors. This is one of the reasons why the optimal strategy for brain protection is controversial. The endogenous factors include haemodynamic instability, dissection of cervical blood vessels, and thromboembolism. In contrast, the extrinsic factors include surgical procedures, such as brain protection strategies, cannulation sites, and embolization due to intraoperative manipulation. Chemtob *et al.* reported that preoperative cerebral malperfusion and impaired haemodynamics were more frequent in patients who developed stroke [2]. In this study, of 42 patients who had postoperative stroke, 27 (64%) presented with preoperative neurological deficits, 21 (50%) with haemodynamic instability, and 10 (24%) with cardiopulmonary resuscitation. In contrast, the percentages significantly differed among the 316 patients who did not have postoperative stroke (22%, *n* = 70; 19%, *n* = 60; and 4%, *n* = 11). In brief, postoperative stroke factors are more likely to be determined by preoperative status than by intraoperative manipulations such as cerebral protection.

Our policy is to accept all patients at any time and never to refuse an emergency operation, even in people of old age or those with poor condition, except for cases of unsuccessful resuscitation or patient refusal. Inevitably, there are more high-risk cases (i.e. a greater risk of postoperative stroke). Nevertheless, the results of this study are comparable to or exceed those of recent databases in terms of mortality and postoperative stroke outcomes, which confirms the safety of brain-protective strategies for MHCA only, without cerebral perfusion.

However, this study had several limitations. First, this was a retrospective, nonrandomized, single-centre study with inherent biases. Second, inadequate brain protection might have caused transient neurological dysfunction, postoperative delirium, confusion, agitation and a decreased level of consciousness, even if no new structural abnormalities are detected on imaging. This study did not examine things that did not appear in the images. Third, as mentioned above, oesophageal temperature best reflects brain temperature, and the use of pharyngeal temperature is recommended. However, the tympanic temperature was used in this study. Finally, although statistically adjusted, the evaluation may not reflect the efficacy of cerebral protection because the effect of preoperative status is considerably greater than that of cerebral protection. To complete the results of this study, more comprehensive pre- and postoperative data as well as a larger sample size are needed. These results can generate hypotheses. Nevertheless, they should be further validated.

## CONCLUSION

This study indicated that MHCA alone for TAAD had comparable postoperative outcomes with circulatory arrest times ≥ and <15 min, which was time previously considered safe. However, longer arrest times were associated with a higher risk of stroke.

## Data Availability

The data supporting the findings of this study will be available from the corresponding author upon reasonable request.

## ABBREVIATIONS

ACP: Antegrade cerebral perfusion
CPB: Cardiopulmonary bypass
DHCA: Deep hypothermia circulatory arrest
HCA: Hypothermia circulatory arrest
MHCA: Moderate hypothermia circulatory arrest
RCP: Retrograde cerebral perfusion
SIRS: Systemic inflammatory response syndrome
TAAD: Type A aortic dissection
